# Correlation of homocysteine with the risk of all-cause mortality in patients with coronary heart disease

**DOI:** 10.3389/fcvm.2025.1624847

**Published:** 2025-09-03

**Authors:** Tianyi Wang, Ruowei Li, Wanqi Gao, Hongting Zhang

**Affiliations:** ^1^ Department of Cardiology, The Second Affiliated Hospital of Heilongjiang University of Chinese Medicine, Harbin, China; ^2^ The Second Clinical College of Heilongjiang University of Traditional Chinese Medicine, Harbin, China; ^3^ Department of Geriatrics, The First Affiliated Hospital of Heilongjiang University of Chinese Medicine, Harbin, China

**Keywords:** homocysteine, coronary heart disease, all-cause mortality, subgroup analysis, sensitivity analysis

## Abstract

**Objective:**

This study aimed to confirm the correlation between homocysteine and all-cause mortality in patients with coronary heart disease (CHD) and to provide new clues and theoretical basis for the improvement of poor prognosis and the development of preventive measures in CHD.

**Methods:**

This single-center retrospective cohort study included 660 patients with CHD. The association between homocysteine and all-cause mortality was assessed using Cox regression analyses, subgroup analyses, sensitivity analyses, receiver operating characteristic curve (ROC), and Kaplan–Meier survival curve.

**Results:**

During a median follow-up time of 45.7 months, 81 all-cause mortality (12%) occurred. Multivariate Cox regression analysis indicated that homocysteine levels were significantly associated with all-cause mortality after adjusting for common confounding factors. Each unit increase and one standard deviation increase in homocysteine levels were associated with a 1% and 15.1% increase in all-cause mortality risk, respectively (HR: 1.010, 95% CI: 1.001–1.019, *P* = 0.038; HR: 1.151, 95% CI: 1.007–1.314, *P* = 0.038). Subgroup analysis showed that the risk of all-cause mortality significantly increased with rising homocysteine levels across multiple subgroups (*P* < 0.05). Sensitivity analysis showed that after excluding patients with chronic kidney disease, atrial fibrillation, prior percutaneous coronary intervention, and myocardial infarction, the multivariable Cox regression analysis still confirmed the robust association between higher homocysteine levels and higher risk of all-cause mortality (*P* < 0.05). ROC analysis showed that homocysteine had a predictive value for the occurrence of all-cause mortality (AUC: 0.660, 95% CI: 0.595–0.726, *P* < 0.001). The Kaplan–Meier survival curve showed that the cumulative risk of all-cause mortality significantly differed between homocysteine groups (Log-rank *P* < 0.001).

**Conclusion:**

Higher levels of homocysteine are significantly associated with a higher risk of all-cause mortality in patients with CHD. This suggests that homocysteine evaluation should be considered in the risk monitoring and prognosis assessment for CHD.

## 1 Introduction

Coronary heart disease (CHD) is a significant cause of morbidity and mortality worldwide, causing serious adverse effects on human health (1). The morbidity and mortality rates of CHD are still on the rise due to the unhealthy lifestyles of Chinese residents, the large population with risk factors for CHD, and the accelerated aging of the population. According to surveys, China currently has about 1.14 billion patients with cardiovascular disease (CVD), of whom about 90,000 cases are CHD patientsand the mortality rate of CHD among Chinese urban residents is 135.08/100,000, and among rural residents is 148.19/100,000 (2). Since 2012, the morbidity and mortality rate of CHD among Chinese urban and rural residents has been on a continuous upward trend, ranking among the highest globally. This public health issue urgently requires attention (3).

In recent years, the incidence of CHD has been on a significant rise due to the continuous influence of multiple risk factors such as high blood pressure, obesity, smoking, poor dietary habits and psychological stress (4–7). These factors interact and superimpose on each other, continuously eroding cardiovascular health, resulting in an increasing risk of CHD and a subtle impact on public health. However, patients with CHD also have a higher incidence of adverse prognosis, including all-cause mortality, after receiving standardized secondary prevention therapy (8, 9). Therefore, early screening and intervention of controllable risk factors for poor prognosis in patients with CHD are of great clinical value and relevance.

Indeed, more than just the traditional risk factors, homocysteine may have some predictive value for the risk of morbidity, severity, and poor prognosis in patients with CHD. Epidemiologic studies have shown that progression and rupture of atherosclerotic plaques are associated with elevated homocysteine levels due to its increased risk of thrombosis, oxidative stress states, and endothelial dysfunction (10). Homocysteine can promote the occurrence, development and occurrence of adverse prognosis of CHD in multiple ways, such as damaging vascular endothelial cells, promoting platelet aggregation, and affecting lipid metabolism (11).

Although the above studies suggest that homocysteine has a contributory role in the occurrence and development of CHD, its correlation with the risk of all-cause mortality in patients with CHD remains under-explored. Therefore, Based on these research backgrounds, the present study aimed to assess the correlation between homocysteine and the risk of all-cause mortality in patients with CHD. Through subgroup analysis, sensitivity analysis, ROC analysis, and Kaplan–Meier survival curve, we will further explore the potential association between them, providing new clues and a theoretical basis for the prevention and treatment measures of CHD.

## 2 Methodology

### 2.1 Study population

This was a single-center retrospective cohort study. This study included 660 patients hospitalized at the Second Affiliated Hospital of Heilongjiang University of Traditional Chinese Medicine between June 1, 2019, and December 31, 2022, for treatment of CHD symptoms, signs, or the need for coronary artery intervention. Inclusion criteria: (1) age ≥18 years old; (2) precise diagnosis of CHD (12) (Including previous myocardial infarction, previous PCI or CABG surgery and positive functional tests, etc.). Exclusion criteria: (1) severe hepatic and renal failure; (2) severe hematologic diseases; (3) malignant tumors; (4) no homocysteine data; (5) loss of visits. After review, 757 people meet the criteria. Among them, 35 people were lost to follow-up due to reasons such as no one answering the phone, so 722 people completed the follow-up. Another 62 people had missing HCY data, resulting in a total of 660 people included in the study. The Ethics Committee of the Second Affiliated Hospital of Heilongjiang University of Traditional Chinese Medicine reviewed and approved the study protocol. Since this was a retrospective study, the Ethics Committee exempted patients from informed consent.

### 2.2 Data collection and definition

In this study, the clinical data of the patients were collected through the electronic medical record system and follow-up records, including baseline information, past history, previous medications, physical examination, and ancillary tests.

Baseline information: age, sex, smoking. Where sex was categorized as male-female and smoking was defined as prolonged past smoking or a quit period of ≤1 year (13). Past medical history: hypertension, diabetes, dyslipidemia, heart failure (HF), myocardial infarction (MI), atrial fibrillation (AF), stroke, chronic kidney disease (CKD), and previous percutaneous coronary intervention (PCI). Hypertension was defined as a previous physician's diagnosis of hypertension or being on antihypertensive medication, or systolic blood pressure (SBP) of ≥140 mmHg or diastolic blood pressure (DBP) of ≥90 mmHg during the current hospitalization (14). Diabetes was defined as a previous physician's diagnosis of diabetes or fasting blood glucose (FBG) ≥7.0 mmol/L or glycated hemoglobin (HbA1c) ≥6.5% during the current hospitalization (15). Dyslipidemia was defined as fasting triglycerides ≥2.3 mmol/L or fasting total cholesterol ≥6.2 mmol/L or fasting low-density lipoprotein cholesterol (LDL-C) ≥4.1 mmol/L during the current hospitalization or fasting high-density lipoprotein cholesterol (HDL-C) <1.0 mmol/L (16). HF was defined as symptoms or signs caused by structural or functional cardiac abnormality confirmed by objective evidence of elevated natriuretic peptide levels and pulmonary or systemic congestion (17). MI was defined as diagnosed by a previous physician or diagnosed according to the guidelines during the current hospitalization (18). AF was defined as a previous physician diagnosis of AF or a guideline-based diagnosis of AF during this hospitalization (19). Moreover stroke was defined as a previous physician diagnosis of stroke or a guideline-based diagnosis of stroke during this hospitalization (20). Prior medications included prior antihypertensive drugs and prior hypoglycemic drugs.

Physical examination: SBP, DBP, heart rate. Blood markers include FBG, HbA1c, triglycerides, total cholesterol, LDL-C, HDL-C, ApoA1, ApoB, lipoprotein(a), uric acid, estimated glomerular filtration rate (eGFR), albumin, homocysteine, high-sensitivity C-reactive protein (Hs-CRP), fibrinogen, D-dimer, high-sensitivity Troponin I (Hs-TnI), and creatine kinase-MB (CK-MB). Ancillary tests: left ventricular ejection fraction (LVEF). Medications used in the hospital included aspirin, clopidogrel, beta-blockers, angiotensin-converting enzyme inhibitors (ACEI), angiotensin-2 receptor antagonists (ARB), calcium channel blockers (CCB), statins, and discharged hypoglycemic drugs.

### 2.3 Measurement and assessment of homocysteine

Measurement method: All the patients in this study ate a light diet, prohibited smoking and drinking alcohol before blood collection, and then went to the laboratory of the First Affiliated Hospital of Heilongjiang University of Chinese Medicine in the morning on an empty stomach (fasting for more than 8 h) to have 3 ml of peripheral venous blood drawn by the staff, and then the plasma homocysteine value was determined by using a dry fluorescent immunoassay analyzer of the model m-20 produced by Shenzhen Micropoint BioTechnology Co, Ltd, and the reference range was 5–15 μmol/L. In this study, homocysteine was analyzed not only as a continuous variable, but also as a categorical variable divided into two groups based on its median (14.1 μmol/L): low homocysteine group (≤14.1 μmol/L) and high homocysteine group (>14.1 μmol/L). It was standardized to standardized homocysteine = (homocysteine raw value—mean value)/standard deviation to better analyze the correlation with the risk of all-cause mortality.

### 2.4 Follow-up and outcomes

All patients in this study were followed up by telephone or previous electronic medical record systems, and follow-up began when the patient was discharged from the hospital and continued until death or April 30, 2025. The follow-up outcome was all-cause mortality, which was defined as death from any cause.

### 2.5 Statistical methods

In this study, there were no missing values for categorical variables. Continuous variables with more than 30% missing values were deleted, and those with less than 30% missing values were imputed using the mean value. All analyses were performed using SPSS 26.0, a two-sided *P* < 0.05 was considered statistically significant. The Shapiro–Wilk normality test assessed all continuous variables for normality before proceeding to the analysis. Continuous variables that did not conform to the normal distribution were expressed as medians (quartiles), and differences between the two groups were tested using the Mann–Whitney *U* test. Categorical variables were expressed as frequencies (percentages), and the chi-square test was used for all differences between the two groups. First, univariate Cox regression analysis was used to assess the correlates of all-cause mortality. Then, three models were constructed by selecting the variables with *P* < 0.05, and multivariate Cox regression analysis was performed. Model 1 was adjusted only for age and sex; Model 2 further adjusted smoking, MI, AF, CKD, HF and stroke based on Model 1; and Model 3 was further adjusted for heart rate, total cholesterol, LDL-C, ApoA1, ApoB, uric acid, eGFR, albumin, fibrinogen, LVEF, β-blocker, and hypoglycemic drugs based on Model 2. Then, fourteen subgroups of age (≤65 years or >65 years), sex (male or female), smoking (yes or no), hypertension (yes or no), diabetes (yes or no), dyslipidemia (yes or no), and stroke (yes or no) were selected for subgroup analyses to assess multivariate stratified associations of homocysteine with all-cause mortality. Patients with CKD, AF, prior PCI, and MI were excluded separately from sensitivity analyses to further explore the robustness of the association between homocysteine and all-cause mortality. On the other side, the predictive value of homocysteine for the occurrence of all-cause mortality was assessed using receiver operating characteristic curve (ROC) analysis. Finally, the Kaplan–Meier survival curve was used to assess the cumulative risk of all-cause mortality differences between homocysteine subgroups.

## 3 Results

### 3.1 Baseline characteristics of all-cause mortality subgroups

As shown in Table 1, the total population was 660, with 81 (12%) all-cause mortality. Compared with no all-cause mortality, the all-cause mortality group had higher age, more men, smokers, and a higher prevalence of HF, MI, AF, stroke, and CKD, as well as higher levels of heart rate, uric acid, homocysteine, Hs-CRP, fibrinogen, and D-dimer, and higher rates of β-blocker, and discharge hypoglycemic drugs use. However, the all-cause mortality group had lower total cholesterol, LDL-C, HDL-C, ApoA1, ApoB, eGFR, albumin, and LVEF (*P* < 0.05).

**Table 1:** Baseline characteristics according to the grouping of all-cause mortality.

Variables	Total population	Non-all-cause mortality	All-cause mortality	*P*-value
*N*	660	579	81	
Age, years	68.00 (61.00, 74.00)	67.00 (60.00, 72.00)	78.00 (70.50, 82.00)	<0.001
Sex, *n* (%)				0.003
Male	230 (34.8)	190 (32.8)	40 (49.4)	
Female	430 (65.2)	389 (67.2)	41 (50.6)	
Smoking, *n* (%)	372 (56.4)	314 (54.2)	58 (71.6)	0.003
Hypertension, *n* (%)	448 (67.9)	393 (67.9)	55 (67.9)	0.996
Diabetes, *n* (%)	222 (33.6)	189 (32.6)	33 (40.7)	0.148
Dyslipidemia, *n* (%)	228 (34.5)	203 (35.1)	25 (30.9)	0.457
Heart failure, *n* (%)	46 (7.0)	30 (5.2)	16 (19.8)	<0.001
Myocardial infarction, *n* (%)	22 (3.3)	12 (2.1)	10 (12.3)	<0.001
Atrial fibrillation, *n* (%)	31 (4.7)	22 (3.8)	9 (11.1)	0.008
Stroke, *n* (%)	260 (39.4)	220 (38.0)	40 (49.4)	0.049
Chronic kidney disease, *n* (%)	82 (12.4)	57 (9.8)	25 (30.9)	<0.001
Previous PCI, *n* (%)	39 (5.9)	31 (5.4)	8 (9.9)	0.127
Antihypertensive drugs, *n* (%)	162 (24.5)	136 (23.5)	26 (32.1)	0.092
Hypoglycemic drugs, *n* (%)	342 (51.8)	298 (51.5)	44 (54.3)	0.630
SBP, mmHg	134.00 (123.00, 148.00)	134.00 (124.00, 148.00)	135.00 (120.00, 148.50)	0.576
DBP, mmHg	80.00 (73.00, 89.00)	80.00 (73.00, 89.00)	80.00 (70.50, 90.50)	0.561
Heart rate, times/min	74.00 (68.00, 81.00)	74.00 (68.00, 80.00)	76.00 (68.00, 87.50)	0.044
Fasting blood glucose, mmol/L	5.51 (4.86, 6.44)	5.49 (4.85, 6.40)	5.66 (4.94, 6.72)	0.418
Glycosylated hemoglobin, %	6.82 (6.10, 6.82)	6.82 (6.10, 6.82)	6.82 (6.20, 6.82)	0.495
Triglycerides, mmol/L	1.36 (1.03, 1.91)	1.36 (1.03, 1.92)	1.28 (1.01, 1.84)	0.212
Total cholesterol, mmol/L	4.96 (4.17, 5.76)	5.05 (4.21, 5.79)	4.54 (3.66, 5.28)	<0.001
LDL-C, mmol/L	2.72 (2.18, 3.32)	2.77 (2.20, 3.38)	2.43 (1.87, 3.00)	0.001
HDL-C, mmol/L	1.41 (1.20, 1.68)	1.42 (1.21, 1.69)	1.33 (1.11, 1.57)	0.031
Apolipoprotein A1, g/L	1.29 (1.10, 1.47)	1.30 (1.11, 1.47)	1.19 (1.02, 1.44)	0.017
Apolipoprotein B, g/L	0.92 (0.72, 1.11)	0.93 (0.73, 1.12)	0.85 (0.66, 1.05)	0.018
Lipoprotein(a), mg/L	235.60 (118.05, 235.60)	235.60 (115.2, 235.60)	235.60 (144.60, 235.60)	0.303
Uric acid, mmol/L	295.00 (242.00, 362.75)	290.40 (239.00, 354.00)	352.00 (271.50, 437.55)	<0.001
eGFR, ml/min/1.73 m^2^	87.22 (71.21, 106.83)	90.38 (73.90, 108.79)	69.54 (55.52, 84.21)	<0.001
Albumin, g/L	41.90 (38.90, 44.30)	42.05 (39.50, 44.40)	39.10 (35.95, 42.45)	<0.001
Homocysteine, μmol/L	14.10 (11.00, 18.30)	13.60 (10.80, 17.80)	16.70 (13.95, 23.65)	<0.001
High-sensitivity C-reactive protein, mg/L	2.80 (1.51, 6.54)	2.70 (1.44, 5.68)	5.05 (2.00, 12.30)	0.001
Fibrinogen, g/L	2.88 (2.45, 3.43)	2.86 (2.43, 3.39)	3.08 (2.52, 3.69)	0.022
D dimer, mg/L	0.40 (0.20, 1.02)	0.37 (0.20, 0.88)	0.78 (0.32, 2.34)	<0.001
High-sensitivity troponin I, ng/ml	0.04 (0.02, 0.05)	0.04 (0.02, 0.05)	0.04 (0.02, 0.05)	0.968
Creatine kinase-MB, U/L	11.00 (9.00, 13.20)	11.00 (9.00, 13.20)	11.60 (9.10, 14.00)	0.172
LVEF, %	65.56 (62.00, 71.00)	66.00 (63.00, 71.00)	65.56 (56.00, 69.50)	0.002
Aspirin, *n* (%)	356 (53.9)	309 (53.4)	47 (58.0)	0.431
Clopidogrel, *n* (%)	137 (20.8)	118 (20.4)	19 (23.5)	0.522
*β*-blocker, *n* (%)	275 (41.7)	230 (39.7)	45 (55.6)	0.007
ACEI/ARB, *n* (%)	195 (29.5)	163 (28.2)	32 (39.5)	0.036
CCB, *n* (%)	249 (37.7)	213 (36.8)	36 (44.4)	0.183
Statins, *n* (%)	482 (73.0)	422 (72.9)	60 (74.1)	0.821
Discharge hypoglycemic drugs, *n* (%)	174 (26.4)	145 (25.0)	29 (35.8)	0.040

PCI, percutaneous coronary intervention; SBP, systolic blood pressure; DBP, diastolic blood pressure; LDL-C, low-density lipoprotein cholesterol; HDL-C, high-density lipoprotein cholesterol; eGFR, estimated glomerular filtration rate; LVEF, left ventricular ejection fraction; ACEI, angiotensin-converting enzyme inhibitors; ARB, angiotensin receptor blockers; CCB, calcium channel blocker.

### 3.2 Baseline characteristics of median homocysteine subgroups

As shown in Table 2, there were two groups based on median homocysteine (14.1 μmol/L): low homocysteine (*n* = 331) and high homocysteine (*n* = 329). Compared with the low homocysteine group, the high homocysteine group had higher age, more men, more smokers, higher rates of hypertension, diabetes, HF, MI, stroke, CKD, prior PCI, and higher antihypertensive drugs use (*P* < 0.05), and the high homocysteine group also had higher levels of uric acid, Hs-CRP, and higher rates of clopidogrel, ACEI, ARB, CCB, and discharge hypoglycemic drugs use (*P* < 0.05). However, the high homocysteine group also had lower levels of total cholesterol, LDL-C, ApoA1, eGFR, albumin, and LVEF (*P* < 0.05).

**Table 2:** Baseline characteristics grouped according to median HCY levels.

Variables	Low HCY	High HCY	*P*-value
Age, years	66.00 (59.00, 71.00)	69.00 (64.00, 78.00)	<0.001
Sex, *n* (%)			<0.001
Male	83 (25.1)	147 (44.7)	
Female	248 (74.9)	182 (55.3)	
Smoking, *n* (%)	160 (48.3)	212 (64.4)	<0.001
Hypertension, *n* (%)	203 (61.3)	245 (74.5)	<0.001
Diabetes, *n* (%)	98 (29.6)	124 (37.7)	0.028
Dyslipidemia, *n* (%)	112 (33.8)	116 (35.3)	0.701
Heart failure, *n* (%)	12 (3.6)	34 (10.3)	0.001
Myocardial infarction, *n* (%)	4 (1.2)	18 (5.5)	0.002
Atrial fibrillation, *n* (%)	11 (3.3)	20 (6.1)	0.094
Stroke, *n* (%)	115 (34.7)	145 (44.1)	0.014
Chronic kidney disease, *n* (%)	9 (2.7)	73 (22.2)	<0.001
Previous PCI, *n* (%)	13 (3.9)	26 (7.9)	0.030
Antihypertensive drugs, *n* (%)	148 (44.7)	194 (59.0)	<0.001
Hypoglycemic drugs, *n* (%)	73 (22.1)	89 (27.1)	0.136
SBP, mmHg	133.00 (122.00, 147.00)	135.00 (123.00, 149.00)	0.315
DBP, mmHg	80.00 (73.00, 89.00)	80.00 (73.00, 90.00)	0.661
Heart rate, times/min	74.00 (68.00, 80.00)	75.00 (69.00, 82.00)	0.260
Fasting blood glucose, mmol/L	5.54 (4.85, 6.22)	5.48 (4.87, 6.83)	0.348
Glycosylated hemoglobin, %	6.82 (6.00, 6.82)	6.82 (6.20, 6.82)	0.090
Triglycerides, mmol/L	1.37 (1.02, 1.90)	1.33 (1.05, 1.96)	0.980
Total cholesterol, mmol/L	5.08 (4.20, 5.81)	4.86 (4.14, 5.67)	0.042
LDL-C, mmol/L	2.80 (2.22, 3.45)	2.66 (2.10, 3.23)	0.027
HDL-C, mmol/L	1.41 (1.21, 1.69)	1.40 (1.18, 1.68)	0.544
Apolipoprotein A1, g/L	1.33 (1.15, 1.49)	1.25 (1.05, 1.44)	<0.001
Apolipoprotein B, g/L	0.92 (0.75, 1.11)	0.92 (0.71, 1.12)	0.560
Lipoprotein(a), mg/L	235.60 (117.60, 235.60)	235.60 (121.45, 235.60)	0.506
Uric acid, mmol/L	282.00 (232.50, 331.50)	318.00 (262.50, 386.50)	<0.001
eGFR, ml/min/1.73 m^2^	97.33 (81.80, 113.71)	78.59 (63.20, 94.42)	<0.001
Albumin, g/L	42.10 (39.10, 44.40)	41.60 (38.30, 44.00)	0.042
High-sensitivity C-reactive protein, mg/L	2.59 (1.43, 5.50)	3.15 (1.56, 7.70)	0.010
Fibrinogen, g/L	2.94 (2.46, 3.43)	2.82 (2.43, 3.40)	0.426
D dimer, mg/L	0.34 (0.19, 0.93)	0.46 (0.23, 1.08)	0.069
High-sensitivity troponin I, ng/ml	0.04 (0.02, 0.05)	0.04 (0.02, 0.05)	0.035
Creatine kinase-MB, U/L	11.00 (9.00, 13.20)	11.10 (9.00, 13.60)	0.864
LVEF, %	66.00 (63.00, 72.00)	65.67 (60.00, 70.00)	<0.001
Aspirin, *n* (%)	177 (53.5)	179 (54.4)	0.810
Clopidogrel, *n* (%)	54 (16.3)	83 (25.2)	0.005
β-blocker, *n* (%)	130 (39.3)	145 (44.1)	0.211
ACEI/ARB, *n* (%)	82 (24.8)	113 (34.3)	0.007
CCB, *n* (%)	103 (31.1)	146 (44.4)	<0.001
Statins, *n* (%)	236 (71.3)	246 (74.8)	0.315
Discharge hypoglycemic drugs, *n* (%)	75 (22.7)	99 (30.1)	0.030

HCY, homocysteine; PCI, percutaneous coronary intervention; SBP, systolic blood pressure; DBP, diastolic blood pressure; LDL-C, low-density lipoprotein cholesterol; HDL-C, high-density lipoprotein cholesterol; eGFR, estimated glomerular filtration rate; LVEF, left ventricular ejection fraction; ACEI, angiotensin-converting enzyme inhibitors; ARB, angiotensin receptor blockers; CCB, calcium channel blocker.

### 3.3 Univariate cox regression analysis of all-cause mortality

As shown in Table 3, univariate Cox regression analysis showed that age, male, smoking, HF, MI, AF, stroke, CKD, heart rate, total cholesterol, LDL-C, ApoA1, ApoB, uric acid, eGFR, albumin, homocysteine, fibrinogen, LVEF, β-blocker, and discharge hypoglycemic drugs were all significantly associated with all-cause mortality risk (*P* < 0.05), with a 1.5% increase in all-cause mortality risk per unit increase in homocysteine (HR: 1.015, 95% CI: 1.008–1.021, *P* < 0.001).

**Table 3:** Univariate Cox regression analysis of all-cause mortality.

Variables	HR	95% CI	*P*-value
Age	1.120	1.092–1.150	<0.001
Male	1.919	1.241–2.966	0.003
Smoking	2.041	1.259–3.308	0.004
Hypertension	1.017	0.638–1.622	0.943
Diabetes	1.393	0.894–2.170	0.143
Dyslipidemia	0.825	0.515–1.322	0.424
Heart failure	3.797	2.197–6.562	<0.001
Myocardial infarction	4.956	2.555–9.612	<0.001
Atrial fibrillation	2.919	1.459–5.839	0.002
Stroke	1.568	1.014–2.424	0.043
Chronic kidney disease	3.609	2.252–5.785	<0001
Previous PCI	1.893	0.912–3.929	0.087
Antihypertensive drugs	1.127	0.728–1.744	0.593
Hypoglycemic drugs	1.537	0.964–2.452	0.071
SBP	0.997	0.986–1.008	0.580
DBP	0.995	0.976–1.013	0.570
Heart rate	1.021	1.006–1.037	0.007
Fasting blood glucose	1.027	0.955–1.104	0.472
Glycosylated hemoglobin	0.966	0.836–1.117	0.642
Triglycerides	0.947	0.753–1.189	0.637
Total cholesterol	0.706	0.583–0.856	<0.001
LDL-C	0.628	0.478–0.826	0.001
HDL-C	0.597	0.325–1.095	0.096
Apolipoprotein A1	0.371	0.160–0.859	0.021
Apolipoprotein B	0.356	0.165–0.764	0.008
Lipoprotein(a)	1.000	0.999–1.001	0.868
Uric acid	1.005	1.003–1.007	<0.001
eGFR	0.971	0.962–0.979	<0.001
Albumin	0.906	0.867–0.946	<0.001
Homocysteine	1.015	1.008–1.021	<0.001
High-sensitivity C-reactive protein	1.000	0.998–1.001	0.813
Fibrinogen	1.290	1.035–1.608	0.023
D dimer	1.000	1.000–1.000	0.922
High-sensitivity troponin I	0.701	0.070–7.061	0.763
Creatine kinase-MB	1.001	0.987–1.015	0.906
LVEF	0.955	0.936–0.975	<0.001
Aspirin	1.154	0.742–1.795	0.524
Clopidogrel	1.121	0.670–1.875	0.663
β-blocker	1.762	1.137–2.731	0.011
ACEI/ARB	1.543	0.988–2.410	0.056
CCB	1.338	0.863–2.075	0.192
Statins	1.021	0.621–1.678	0.935
Discharge hypoglycemic drugs	1.633	1.037–2.573	0.034

PCI, percutaneous coronary intervention; SBP, systolic blood pressure; DBP, diastolic blood pressure; LDL-C, low-density lipoprotein cholesterol; HDL-C, high-density lipoprotein cholesterol; eGFR, estimated glomerular filtration rate; LVEF, left ventricular ejection fraction; ACEI, angiotensin-converting enzyme inhibitors; ARB, angiotensin receptor blockers; CCB, calcium channel blocker.

### 3.4 Multivariate Cox regression analysis of homocysteine and all-cause mortality

As shown in Table 4, in Model 1 adjusted only for age and sex, the increase in homocysteine levels was significantly correlated with the risk of all-cause mortality. The risk of all-cause mortality increased by 1% and 15% for each unit and standard deviation increase in homocysteine, respectively (HR: 1.010, 95% CI: 1.002–1.018, *P* = 0.017; HR: 1.150, 95% CI: 1.026–1.290, *P* = 0.017), and the risk of all-cause mortality in the high homocysteine group had 1.728 times the risk of all-cause mortality than the low homocysteine group (HR: 1.728, 95% CI: 1.036–2.885, *P* = 0.036). In Model 2, which adjusted for age, sex, smoking, MI, AF, CKD, HF, and stroke, the increase in homocysteine is still significantly associated with the occurrence of all-cause mortality. The risk of all-cause mortality increased by 1.2% and 18.5% for each unit and standard deviation increase in homocysteine, respectively (HR: 1.012, 95% CI: 1.003–1.021, *P* = 0.007; HR: 1.185, 95% CI: 1.048–1.341, *P* = 0.007). Additionally. In Model 3, which adjusted for heart rate, total cholesterol, LDL-C, ApoA1, ApoB, uric acid, eGFR, albumin, fibrinogen, LVEF, β-blockers, and discharge hypoglycemic drugs based on Model 2, homocysteine as a continuous variable was still significantly associated with the risk of all-cause mortality, where for each unit and standard deviation increase in homocysteine, the all-cause mortality risk increased by 1% and 15.1%, respectively (HR: 1.010, 95% CI: 1.001–1.019, *P* = 0.038; HR: 1.151, 95% CI: 1.007–1.314, *P* = 0.038).

**Table 4:** Multivariate Cox regression analysis of HCY and all-cause mortality.

Variables	Model 1			Model 2			Model 3		
	HR	95% CI	*P*-value	HR	95% CI	*P*-value	HR	95% CI	*P*-value
HCY	1.010	1.002–1.018	0.017	1.012	1.003–1.021	0.007	1.010	1.001–1.019	0.038
Standardized HCY	1.150	1.026–1.290	0.017	1.185	1.048–1.341	0.007	1.151	1.007–1.314	0.038
Low HCY	Ref			Ref			Ref		
High HCY	1.728	1.036–2.885	0.036	1.516	0.875–2.629	0.138	1.295	0.739–2.272	0.366

Model 1: Only adjusted for age and sex; Model 2: Adjusted for age, sex, smoking, myocardial infarction, atrial fibrillation, chronic kidney disease, heart failure and stroke; Model 3: Adjusted for age, sex, smoking, myocardial infarction, atrial fibrillation, chronic kidney disease, heart failure, stroke, heart rate, total cholesterol, LDL-C, apolipoprotein A1, apolipoprotein B, uric acid, eGFR, albumin, fibrinogen, LVEF, β-blocker and discharge hypoglycemic drugs.

LDL-C, low-density lipoprotein cholesterol; eGFR, estimated glomerular filtration rate; LVEF, left ventricular ejection fraction; HCY, homocysteine; HR, hazard ratio; CI, confidence interval.

### 3.5 Subgroup analysis of homocysteine with all-cause mortality

As shown in Table 5, participants were divided into 14 subgroups based on age (≤65 years or >65 years), gender (male or female), smoking status (yes or no), hypertension (yes or no), diabetes (yes or no), dyslipidemia (yes or no), and stroke (yes or no), to further analyze the association differences of homocysteine levels across these subgroups. The risk of all-cause mortality was significantly increased in multiple subgroups, including age ≤65 years or >65 years, male, smoking, without hypertension, without diabetes, without dyslipidemia, and with stroke (*P* < 0.05).

**Table 5:** Subgroup analyses of the correlation between HCY and all-cause mortality.

Subgroups	HCY			Standardized HCY			High HCY vs. low HCY		
	HR	95% CI	*P*-value	HR	95% CI	*P*-value	HR	95% CI	*P*-value
Age									
≤65 years	1.077	1.015–1.142	0.014	2.899	1.237–6.797	0.014	0.093	0.011–0.801	0.031
>65 years	1.012	1.002–1.002	0.020	1.181	1.026–1.360	0.020	1.693	0.895–3.200	0.105
Sex									
Male	1.340	1.088–1.651	0.006	1.145	1.040–1.261	0.006	2.835	1.150–6.987	0.024
Female	1.005	0.989–1.022	0.515	1.080	0.857–1.362	0.515	0.702	0.314–1.571	0.390
Smoking									
Yes	1.025	1.011–1.039	0.001	1.425	1.163–1.745	0.001	2.029	1.000–4.116	0.050
No	1.010	0.991–1.029	0.305	1.151	0.880–1.506	0.305	0.324	0.096–1.088	0.068
Hypertension									
Yes	1.015	0.994–1.037	0.167	1.240	0.914–1.683	1.167	1.128	0.562–2.264	0.735
No	1.015	1.002–1.029	0.019	1.247	1.036–1.501	0.019	1.204	0.360–4.029	0.763
Diabetes									
Yes	0.929	0.856–1.007	0.074	0.343	0.106–1.110	0.074	0.821	0.295–2.285	0.705
No	1.011	1.002–1.020	0.013	1.172	1.035–1.328	0.013	1.531	0.729–3.214	0.261
Dyslipidemia									
Yes	0.943	0.864–1.028	0.181	0.426	0.122–1.490	0.181	0.463	0.166–1.290	0.141
No	1.017	1.008–1.026	<0.001	1.271	1.114–1.451	<0.001	1.832	0.892–3.765	0.099
Stroke									
Yes	1.018	1.006–1.029	0.003	1.289	1.092–1.521	0.003	1.150	0.513–2.578	0.733
No	1.006	0.990–1.021	0.480	1.084	0.867–1.356	0.480	1.151	0.488–2.714	0.747

The subgroup analysis adjusted for age, sex, smoking, myocardial infarction, atrial fibrillation, chronic kidney disease, heart failure, stroke, heart rate, total cholesterol, LDL-C, apolipoprotein A1, apolipoprotein B, uric acid, eGFR, albumin, fibrinogen, LVEF, beta-blockers and hypoglycemic drugs.

LDL-C, low-density lipoprotein cholesterol; eGFR, estimated glomerular filtration rate; LVEF, left ventricular ejection fraction; HCY, homocysteine; HR, hazard ratio; CI, confidence interval.

### 3.6 Sensitivity analysis of the correlation between homocysteine and all-cause mortality

As shown in Table 6, higher levels of homocysteine were still significantly associated with a higher risk of all-cause mortality after excluding patients with CKD, AF, prior PCI, or MI. Multivariate Cox regression analyses showed that for each one-unit and one-standard deviation increase in homocysteine, the risk of all-cause mortality increased by 1.2% and 19%, respectively, after excluding patients with CKD (HR: 1.012, 95% CI: 1.003–1.021, *P* = 0.010; HR: 1.190, 95% CI: 1.043–1.357, *P* = 0.010); after excluding patients with AF, the risk of all-cause mortality increased by 1% and 15.5% for each unit and standard deviation increase in homocysteine (HR: 1.010, 95% CI: 1.000–1.020, *P* = 0.042; HR: 1.155, 95% CI: 1.006–1.326, *P* = 0.042).); for each unit and standard deviation increase in homocysteine, the risk of all-cause mortality increased by 1.5% and 24%, respectively, after excluding patients with prior PCI (HR: 1.015, 95% CI: 1.006–1.024, *P* = 0.002; HR: 1.240, 95% CI: 1.085–1.417, *P* = 0.002); furthermore, after excluding patients with MI, a one-unit and one-standard-deviation increase in homocysteine was still associated with a significantly increased risk of all-cause mortality of 1.1% and 17.7%, respectively (HR: 1.011, 95% CI: 1.003–1.020, *P* = 0.011; HR: 1.177, 95% CI: 1.039–1.335, *P* = 0.011).

**Table 6:** Sensitivity analysis: exclusion of patients with CKD, AF, prior PCI, or MI, respectively.

Variables	Model 1			Model 2			Model 3		
	HR	95% CI	*P*-value	HR	95% CI	*P*-value	HR	95% CI	*P*-value
Exclusion of CKD									
HCY	1.010	1.001–1.018	0.023	1.013	1.004–1.022	0.005	1.012	1.003–1.021	0.010
Standardized HCY	1.149	1.019–1.295	0.023	1.089	1.058–1.366	0.005	1.190	1.043–1.357	0.010
Low HCY	Ref			Ref			Ref		
High HCY	1.439	0.831–2.492	0.194	1.515	0.849–2.704	0.160	1.333	0.735–2.418	0.345
Exclusion of AF									
HCY	1.010	1.002–1.018	0.020	1.012	1.003–1.021	0.010	1.010	1.000–1.020	0.042
Standardized HCY	1.151	1.023–1.295	0.020	1.184	1.041–1.346	0.010	1.155	1.006–1.326	0.042
Low HCY	Ref			Ref			Ref		
High HCY	1.849	1.063–3.216	0.030	1.488	0.826–2.680	0.186	1.327	0.731–2.408	0.353
Exclusion of prior PCI									
HCY	1.012	1.004–1.021	0.003	1.015	1.006–1.024	0.001	1.015	1.006–1.024	0.002
Standardized HCY	1.195	1.062–1.344	0.003	1.237	1.091–1.402	0.001	1.240	1.085–1.417	0.002
Low HCY	Ref			Ref			Ref		
High HCY	1.688	0.991–2.873	0.054	1.405	0.793–2.491	0.244	1.254	0.700–2.249	0.447
Exclusion of MI									
HCY	1.010	1.003–1.018	0.009	1.012	1.004–1.021	0.003	1.011	1.003–1.020	0.011
Standardized HCY	1.163	1.038–1.303	0.009	1.196	1.064–1.346	0.003	1.177	1.039–1.335	0.011
Low HCY	Ref			Ref			Ref		
High HCY	1.735	1.009	2.982	1.615	0.909–2.869	0.102	1.419	0.791–2.546	0.241

Model 1: Adjusted only for age and sex; Model 2: Adjusted for age, sex, smoking, myocardial infarction, atrial fibrillation, chronic kidney disease, heart failure and stroke; Model 3: Adjusted for age, sex, smoking, myocardial infarction, atrial fibrillation, chronic kidney disease, heart failure, stroke, heart rate, total cholesterol, LDL-C, apolipoprotein A1, apolipoprotein B, uric acid, eGFR, albumin, fibrinogen, LVEF, β-blocker and hypoglycemic drugs.

CKD, chronic kidney disease; AF, atrial fibrillation; PCI, percutaneous coronary intervention; MI, myocardial infarction; LDL-C, low-density lipoprotein cholesterol; eGFR, estimated glomerular filtration rate; LVEF, left ventricular ejection fraction; HCY, homocysteine; HR, hazard ratio; CI, confidence interval.

### 3.7 The predictive value of homocysteine for all-cause mortality

**Figure 1:**
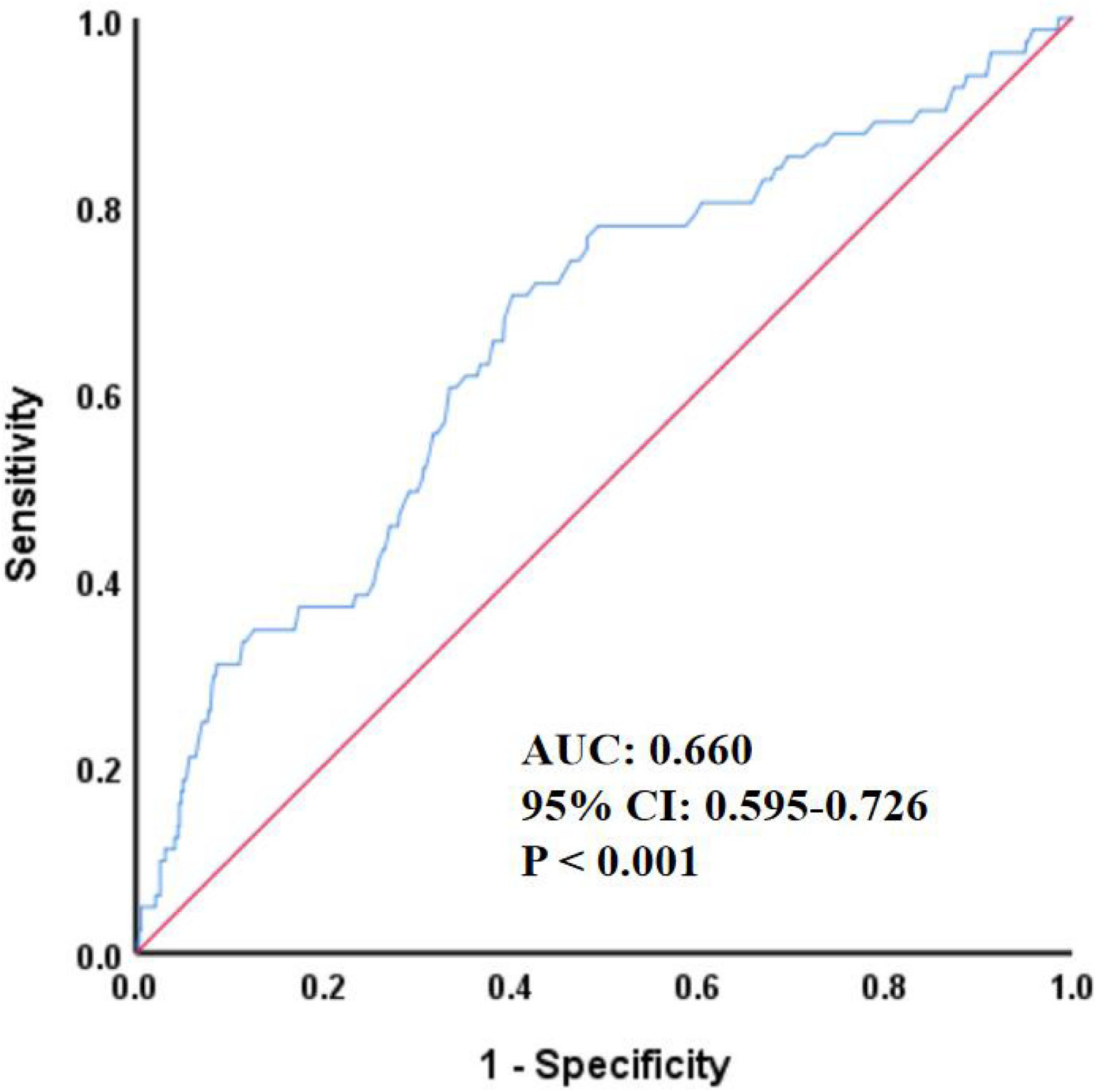
ROC curve for predicting all-cause mortality by HCY. HCY, homocysteine; ROC, receiver operating characteristic; AUC, area under the curve; CI, confidence interval.

As shown in Figure 1, ROC analysis demonstrated that homocysteine could predict the occurrence of all-cause mortality (AUC 0.660, 95% CI 0.595–0.726, *P* < 0.001).

### 3.8 Kaplan–Meier survival curve

**Figure 2:**
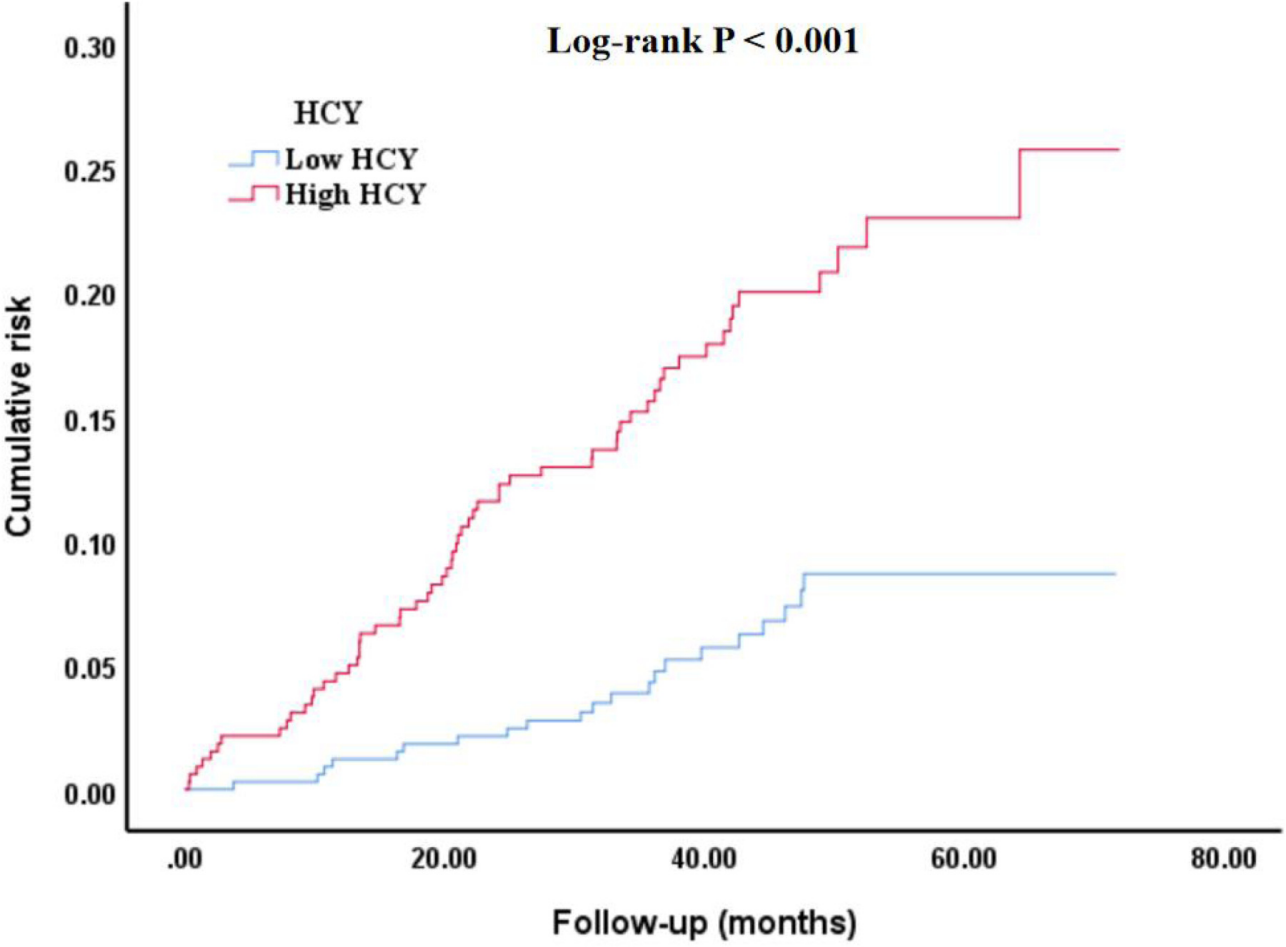
Kaplan–Meier survival curve of cumulative risk of all-cause mortality among different homocysteine groups. HCY, homocysteine.

As shown in Figure 2, the cumulative risk of all-cause mortality was significantly different in different homocysteine groups (Log-rank *P* < 0.001). Among them, the high homocysteine group had greater cumulative risk, worse healing, and higher all-cause mortality.

## 4 Discussion

In this retrospective cohort study based on real-world data, multivariate Cox regression analysis revealed that homocysteine levels remained significantly associated with the risk of all-cause mortality after adjusting for all potential confounding factors, sensitivity analysis, and subgroup analysis. Furthermore, ROC analysis indicated that homocysteine had a specific predictive value for the occurrence of all-cause mortality. On the other hand, the Kaplan–Meier survival curve showed a significant difference in the cumulative risk of all-cause mortality between the homocysteine groups. These findings suggest that homocysteine may have important clinical value in the risk monitoring and prognosis assessment of CHD.

Current evidence suggests that homocysteine is recognized as a novel independent risk factor for a variety of CVD, and its exploration has continued in recent years. For example, in an observational study in recent years, homocysteine was found to be significantly associated with the severity of coronary artery stenosis in young patients with CHD under 35 years of age and, to some extent, predicted the degree of stenosis of coronary arteries (21). In addition, research has found that serum homocysteine and MTHFR gene polymorphism may affect the development of CHD combined with blood diseases. In the future, these two indicators can be tested to monitor the risk of CHD in patients with blood diseases at an early stage (22). Moreover, in another study, elevated levels of homocysteine and Lp-PLA2 are both independent risk factors for CHD, and combined analysis of the two can significantly improve the accuracy of clinical diagnosis, providing an important basis for early screening and prevention of CHD (23). Meanwhile, elevated homocysteine predicts the risk of major adverse cardiovascular events in female preterm infants with acute coronary syndromes, which is of great significance for the detection of adverse cardiovascular events (24). In addition, in a Meta, it was found that for every 22 μmol/L increase in plasma homocysteine levels, the incidence of CHD increased by 5% compared to traditional risk factors. This suggests that we need to pay attention to elevated homocysteine levels, which is important for reducing the incidence of CHD (25). Moreover, high homocysteine levels are associated with severe CHD in patients with CHD after stent implantation, and this study contributes to the risk monitoring of patients with CHD with previous stent implantation (26). In fact, the combined detection of serum neuron-specific enolase and homocysteine is also informative for diagnosing CHD (27). Every discovery of the researchers is an innovative push for the prevention, diagnosis and prognosis of CHD. In order to further explore the correlation between homocysteine and CHD, the present study also made some innovations. The content of both studied the correlation between homocysteine and CHD. However, the difference is that the present study collected more blood markers, past medical history, and medication during hospitalization. Subgroup analysis, sensitivity analysis, ROC analysis, Kaplan–Meier survival curves and other research methods were also used to analyze the correlation between homocysteine and all-cause mortality of patients with CHD under different conditions in a deeper and more comprehensive way.

The present study, despite confirming the correlation between homocysteine and all-cause mortality in patients with CHD, remains poorly informed about the underlying biological mechanisms involved. Homocysteine is a sulfur amino acid produced by the metabolism of methionine, which is metabolized through two key pathways: remethylation to methionine by a series of enzymes when methionine is deficient, and transsulfuration to cysteine when methionine is sufficient (28), which, if dysregulated, may result in elevated plasma homocysteine levels. The normal homocysteine range is 5–15 μmol/L; hyperhomocysteinemia is defined as blood levels >15 μmol/L (29). If the homocysteine level in patients with CHD is elevated, even exceeding 15 μmol/L, it may lead to the occurrence of adverse cardiovascular events. The vascular endothelium is an important barrier to vascular homeostasis. Homocysteine can damage endothelial cells and smooth muscle cells by inducing inflammation and cell death, interfering with NO production, ROS accumulation and oxidative stress, cellular hypomethylation, and other mechanisms, which can lead to vascular endothelial dysfunction and endothelial barrier damage, resulting in the development and progression of atherosclerosis (30). In addition, homocysteine promotes platelet aggregation. Homocysteine can systematically modulate biased G protein-coupled receptor signaling through inhibition of β-arrestin desensitization and amplification of G protein signaling, which is a central regulator of the cardiovascular system that controls platelet activation. This thereby accelerates the progression of atherosclerosis and leads to the occurrence of adverse cardiovascular events such as all-cause mortality (31). Research indicates that elevated homocysteine levels can disrupt lipid metabolic balance. When homocysteine concentrations in the body increase, it promotes the production of S-adenosyl-L-homocysteine, a metabolic byproduct that stimulates enhanced lipid synthesis and accelerates the formation of atherosclerotic plaques. As atherosclerotic plaques continue to accumulate, coronary arteries gradually narrow, ultimately triggering and exacerbating the onset and progression of CHD (32). Another study showed that homocysteine could stimulate the production of CRP to initiate an inflammatory response in vascular smooth muscle cells, which in turn promotes the occurrence and development of CHD, and this finding provides new evidence for the role of homocysteine in the pathogenesis of atherosclerosis (33). It is precisely these mechanisms that enable homocysteine to promote the occurrence and development of CHD and increase the all-cause mortality rate. Therefore, we should attach importance to the value of homocysteine in the risk monitoring and prognosis assessment of CHD, achieve early screening and prevention of the disease, and reduce the incidence of adverse events such as all-cause mortality.

Despite the valuable findings obtained in this study, some limitations remain unavoidable. First, as an observational study, we could not determine the causal association between homocysteine and all-cause mortality in patients with CHD, and genetic association studies will be conducted in the future, based on the availability of conditions, to further determine their causal association. Second, this was a single-center retrospective study with weak research evidence that may not be applicable to other races and populations, and more large-sample multicenter prospective clinical trials are expected to be conducted in the future to further validate their association. Third, although this study included a relatively large number of clinical indicators, it still lacked some important indicators. For example, coronary angiography-related parameters, dietary factors, environmental factors, and genetic susceptibility, which will be further remedied in future studies. Fourth, only one follow-up outcome, all-cause mortality, was included in this study. Since other outcomes, including cardiovascular death, nonfatal MI, nonfatal stroke, and unplanned revascularization, did not show significant associations with homocysteine, further exploration and presentation of results were not performed in this study, and their associations will be verified again in the future by expanding the sample size and extending the follow-up period. Fifth, although this study found that higher levels of homocysteine were significantly associated with a higher risk of all-cause mortality, no significant association was observed when homocysteine was used as a categorical variable. This may be attributed to the small sample size, short follow-up period, and regional limitations of this study. In sensitivity analyses, we also explored different subgroups and found that, in certain subgroups, the high homocysteine group had a higher risk of all-cause mortality compared to the low homocysteine group. Sixth, as shown in Table 4, in the complete adjustment model, the risk of all-cause mortality only increased by about 1% for every one-unit increase in homocysteine. This occurs because the homocysteine value is relatively high, so the impact of each unit change on the mortality rate is also relatively small. Based on this situation, we carried out a standardization process, and the results showed that for every one standard deviation increase in homocysteine, the risk of all-cause mortality increases by 15.1%. Moreover, many studies have also adopted standardization to handle such continuous variables with a wide range of values, making the outcomes more representative. Furthermore, we also divided the subjects into two groups based on the median level of homocysteine. In the fully adjusted model, the results did not indicate a correlation between the high homocysteine group and all-cause mortality. This might be due to the small sample size and the mismatch of the groups. This was also a limitation of this study. In future research, we will further increase the sample size and try different thresholds to explore the their significant association again. Although the ROC curve indicated that homocysteine had a specific predictive value for all-cause mortality, the AUC was only 0.660, which was considered a moderately weak predictive level in ROC analysis. This may also be related to the small sample size in this study. In future studies, we will further increase the sample size to enhance the predictive value of homocysteine for all-cause mortality risk. Seventh, although all hematological indicators for patients included in this study were measured from fasting venous blood during hospitalization, some variables inevitably had missing values, which could affect the accuracy of the study results. Therefore, we deleted continuous variables with missing values exceeding 30% and imputed continuous variables with missing values below 30% to compensate for data missing bias. These limitations inevitably restrict the generalization and application of the results of this study. However, these important findings cannot be denied, and the above shortcomings will be remedied in the future to further improve the study design.

## 5 Conclusion

This single-center retrospective cohort study confirms the significant association of homocysteine with all-cause mortality and subgroup differences in CHD patients. This not only broadens the field of research in CHD risk monitoring and prognostic assessment, but also indicates that homocysteine may be a promising biomarker worthy of further exploration in larger-scale prospective studies.

## Data Availability

The original contributions presented in the study are included in the article/Supplementary Material, further inquiries can be directed to the corresponding author.
